# Seasonal Temperature Differentially Modulates the Immunotranscriptomic Performance in Atlantic Salmon Skin in Response to Natural *Caligus rogercresseyi* Infestation in Open-Ocean Cages

**DOI:** 10.3390/ani15162369

**Published:** 2025-08-12

**Authors:** Andrea Cerda-Celis, Mabel Vidal, Merari Goldstein, Maria Jesús Santillán-Araneda, Alexis Rivera, Daniela Vargas, Gabriel Jerez, Eva Vallejos-Vidal, Sebastian Reyes-Cerpa, Felipe E. Reyes-López

**Affiliations:** 1 Fish Health and Integrative Physiogenomics Research Team, Centro de Biotecnología Acuícola, Facultad de Química y Biología, Universidad de Santiago de Chile, Avenida Libertador Bernardo OHiggins 3363, Edificio de Investigación Eduardo Morales, Santiago 9170002, Chile; andrea.cerda@usach.cl (A.C.-C.); mabvidal@udec.cl (M.V.); merari.goldstein@usach.cl (M.G.); maria.santillan@usach.cl (M.J.S.-A.); arivera.esl@gmail.com (A.R.); evallejos@udla.cl (E.V.-V.); 2 Departamento de Ingeniería Informática y Ciencias de la Computación, Universidad de Concepción, Edmundo Larenas 219, Concepción 4070409, Chile; 3 ANID Millenium Nucleus in Data Science for Plant Resilience (Phytolearning), Santiago 8370186, Chile; 4 Salmones Blumar S.A, Puerto Montt 5504750, Chile; daniela.vargas@blumar.com (D.V.); gabriel.jerez@blumar.com (G.J.); 5 Núcleo de Investigación en Producción y Salud de Especies Acuáticas (NIP-SEA), Facultad de Medicina Veterinaria y Agronomía, Universidad de Las Américas, Avenida Walker Martínez 1360, La Florida, Santiago 8242125, Chile; 6 Centro de Genómica y Bioinformática, Facultad de Ciencias, Ingeniería y Tecnología, Universidad Mayor, Santiago 8580745, Chile; 7 Escuela de Biotecnología, Facultad de Ciencias, Ingeniería y Tecnología, Universidad Mayor, Santiago 8580745, Chile

**Keywords:** Atlantic salmon, *Caligus rogercresseyi*, transcriptomics, seasonal temperature ramp, skin-associated lymphoid tissue, aquaculture

## Abstract

Chile is the second-largest producer of Atlantic salmon (*Salmo salar*), a key industry facing challenges such as infestations by *Caligus rogercresseyi*, which cause significant economic losses. However, there are no studies exploring how environmental variables in marine culture centers affect salmon’s immune response, considering their poikilothermic characteristics. This study analyzed the effect of the seasonal temperature on the skin transcriptome of Atlantic salmon infested with *C. rogercresseyi* in fjord-based farms in the Aysén Region during autumn and spring. Two different centers were analyzed (autumn: Farm-A; spring: Farm-S) to ensure the same age of the fish sampled in each season. RNA sequencing (RNA-seq) and functional network analysis revealed notable seasonal transcriptomic differences. In autumn, 253 differentially expressed genes (DEGs) were found, primarily associated with stress response and negative regulation of T-cell proliferation, suggesting an impaired response against the ectoparasite. In spring, 103 DEGs were detected, associated with protein synthesis and the activation of immune mechanisms, including complement activation, granulocyte chemotaxis, and antigen presentation. This is the first study to evaluate the transcriptomic response of healthy skin in *C. rogercresseyi*-infested salmon under farming conditions, highlighting the importance of considering seasonal variations for the development of more effective management strategies in the aquaculture industry.

## Simple Summary

Atlantic salmon farming is a key economic activity in Chile, but it faces challenges from infestations by *Caligus rogercresseyi*. This parasite causes health problems in fish and economic losses for producers. Since salmon are poikilothermic animals, their immune response can vary with environmental conditions. This study analyzed the seasonal transcriptomic response in salmon skin under farming conditions, revealing distinct patterns between autumn and spring. In autumn, the response was dominated by stress-related pathways, mitochondrial dysfunction and early tissue repair signals. In contrast, spring samples showed activation of both innate and adaptive immune mechanisms, including complement activation, chemotaxis, and antigen presentation. These findings suggest that seasonal temperature variations significantly shape the host response to parasitic challenge and should be considered in the development of effective management strategies in aquaculture.

## 1. Introduction

Atlantic salmon (*Salmo salar* L.), an anadromous species of the Salmonidae family, stands out as one of the main species in global aquaculture, occupying fifth place in world fish production in 2022 with 2.86 million metric tons [1]. Chile, as the world’s second-largest producer of this species, reached a production of 774,531 metric tons in 2023, generating revenues close to USD 6462 million [2]. During 2022, Chilean exports of Atlantic salmon accounted for 7% of the national total, consolidating its position as the most exported industrial food, with a contribution of 1.12% to the country’s gross domestic product, and positioning it as the second-most exported product after copper [3].

Intensive farming has allowed a steady growth in the production of salmon in captivity. However, this growth has not been without challenges, including increased disease and parasite transmission [4]. Infestation by the ectoparasite sea louse (two main genera, Lepeophtheirus and Caligus) stands out as one of the major problems affecting salmon aquaculture globally and nationally [5]. The ectoparasite *C. rogercresseyi* is the only sea louse affecting the Chilean salmon industry and the etiological agent of caligidosis [5]. Additionally, sea louse infestation causes significant economic losses, with an increase of USD 1.37 per kilogram of Atlantic salmon due to the antiparasitic treatments necessary to control the infestation [6]. Likewise, the wounds generated in the skin of Atlantic salmon compromise the integrity and quality of the fillet [7].

The life cycle of *C. rogercresseyi* includes eight developmental stages, three planktonic (two nauplii and one copepodite free-living/infesting stages) and five parasitic (four chalimus and motile adult). In its parasitic state, the sea louse feeds on the mucus, skin, and blood of the fish, causing wounds, stress, weakening, gill damage, and, in some cases, osmoregulatory failures in the host [6,8]. The life cycle of this parasite depends on environmental factors such as water temperature. It has a duration of 45 days at a temperature of 10.3 °C, while the life cycle is shortened to 26 days when the water temperature increases to 15.2 °C [9]. In this sense, it has been described that Atlantic salmon have a wide range of thermal tolerance (−0.8 °C to 33 °C) [10], with their optimal range being between 4 °C and 17.5 °C [11]. However, fluctuations in water temperature outside the optimal range in Atlantic salmon can affect the immune system [12,13], metabolism, phenology, and survival, among others [13,14,15].

Water temperature is also a critical environmental factor for teleost fish such as Atlantic salmon. Unlike mammals, fish cannot regulate their body temperature because of their poikilothermic condition, thus conditioning their metabolism and, consequently, their responsiveness to the aquatic environmental temperature [15]. In Atlantic salmon, it has been observed that the increase in temperature produces an upregulation of genes associated with stress, such as *Hsp70*, and an increase in the production of pro-inflammatory cytokines such as *il-1β* and *tnf-α*, which play a key role in the fight against infections [16]. When the thermal increase exceeds certain limits, this effect becomes detrimental, leading to alterations in the balance of the cutaneous microbiome and a decrease in mucus secretion, which weakens the physical barrier and increases susceptibility to pathogens [16]. On the other hand, lower temperatures tend to reduce metabolic activity and slow down the immune response, which can compromise the effectiveness of defenses against infections [17]. In carp *(Cyprinus carpio)* after infection with *Aeromonas salmonicida* subsp., it was shown that in autumn, the expression of *inos*, *il-12p35*, *ifn-γ2*, and *arginase 2* was increased, while in spring, *inos*, *il-12p35*, *ifn-γ2*, *arginase 2*, and *il-10* were positively regulated, in addition to *crp2* at 96 hpi (hours post-infection), evidencing a seasonally differentiated immune response [18]. On the other hand, a study in *Oncorhynchus mykiss* evaluated the impact of seasonal changes on the humoral and immunological status of blood, simulating field conditions in a laboratory environment. It was observed that the number of leukocytes varied with water temperature, with an increase in peroxidase activity in summer, while lysozyme and antiprotease increased in winter. Additionally, antioxidant defenses exhibited seasonal fluctuations, with an increase in oxidative damage at higher temperatures [19].

The skin mucosa is in direct contact with the water environment, acting as a high-precision physical barrier to preserve its integrity and the survival of organisms [20,21]. The skin mucosa of teleosts is an extensive multilayered integument (cuticle or mucous layer, epidermis, and dermis) that is non-keratinized and metabolically active and produces a mucus layer based on complex glycoproteins called mucins [22]. In teleosts, the SALT harbors a more diverse repertoire of innate humoral components than that of mammals, including bacteriolytic molecules such as lysozyme, complement components, lectins, proteolytic enzymes, C-reactive protein, interferons, and immunoglobulins [23]. A whole cast of resident leukocytes completes the immunological properties of the SALT [24]. Thus, skin mucosa is considered a mucosa-associated lymphoid tissue (MALT) [25,26], also referred to as skin-associated lymphoid tissue (SALT).

The response of Atlantic salmon skin infested with L. salmonis showed an elevated expression of pro-inflammatory mediators (*il-1β*, *il-8*, *tnfα*, *cox2*, *c/ebpβ*) and tissue repair enzymes (*mmp9*, *mmp13*) [27]. The upregulation of pro-inflammatory cytokines in Atlantic salmon infested by *L. salmonis* was associated with a CD4+ T-helper (Th) cell-mediated response. A previous study in Atlantic salmon infested with *C. rogercresseyi* showed a specific immune response involving a Th1 response [5]. However, Coho salmon, a salmonid species with higher resistance to infestation by *L. salmonis* [28,29], showed upregulation of *il-4*, suggesting a role of type 2 immunity in the defense mechanism against the ectoparasite [27,30]. Additionally, a study reported the upregulation of genes associated with tissue repair in Atlantic salmon infested with *C. rogercresseyi* [31].

The contribution of these studies conducted under controlled laboratory conditions contrasts with the lack of studies aimed at characterizing the skin response of Atlantic salmon to *C. rogercresseyi* in fish farms grown under production regimes. Furthermore, it is unknown whether intrinsic variations associated with seasonal temperature modulate the skin’s response to infestation by the ectoparasite. Therefore, this study analyzed the effect of the seasonal temperature on the skin transcriptome of Atlantic salmon infested with *C. rogercresseyi* in fjord-based farms in the Aysén Region during autumn (marked by a gradual thermal decrease from summer to winter) and spring (characterized by a gradual temperature increase from winter to summer). Two different centers were analyzed (autumn: Farm-A; spring: Farm-S) to ensure the same age of the fish sampled in each season. This approach addresses the seasonal host–ectoparasite interaction in the skin response of Atlantic salmon, providing a scientific basis for improving health management strategies in the salmon industry.

## 2. Materials and Methods

### 2.1. Ethical Statement

The experimental procedure was carried out following the ethical guidelines for animal experimentation (“Código de Ética de la Sociedad de Biología de Chile”, 2008). The protocols were authorized by the Institutional Ethics Committee of the Universidad de Santiago de Chile (Ethics Report N° 206.2021).

### 2.2. Fish and Sampling

Atlantic salmon were collected from commercial fish farms under an open-sea cage productive regime located in the fjords of the Aysén Region (the second southernmost region of Chile), the main Atlantic salmon-producing region in Chile. The seasonal temperature effect was analyzed in two different seasons of the year: autumn (marked by a gradual thermal decrease from summer to winter) and spring (characterized by a gradual temperature increase from winter to summer). For this purpose, and in order to ensure a similar age of the fish sampled in each season, two different fish farms were included (autumn: Farm-A; spring: Farm-S). In both cases, fish were transferred to the open-sea cage marine regime in the absence of hexaflumuron treatment (PHARMAQ AS; Overhalla, Norway), which is applied in freshwater prior to transfer to sea to protect fish from caligidosis in the first months in open-sea cages. For “Farm-A”, fish were stocked in open-sea cages in week 49 of 2021 (December 2021) to ensure that they experienced the peak annual maximum temperature and then a progressive decrease in temperature until they were sampled in the autumn (week 20, May 2022). On the other hand, fish from “Farm-S” were stocked in week 24 of 2022 (June 2022) to ensure that they experienced the peak annual minimum temperature, and then the temperature was progressively increased until they were sampled in spring (week 47, November 2022). At the moment of sampling, the water temperature was at 10.5 °C on both farms. This precision was achieved through the periodic, real-time determination of the minimum and maximum water temperatures using sensors located in the water column at depths of 5 m and 10 m (Innovex; Puerto Montt, Chile), in accordance with the Chilean sanitary regulations in force for salmon farming production.

Regarding sampling, fish were lured to the surface with the aid of food and collected using a net. Subsequently, the fish were individually captured and individually arranged in a 200 L tank with water previously collected from the open-sea cage. Fish were immediately inspected by macroscopic visualization to determine the presence (infested fish) (n=15 fish per each farm) or absence of *C. rogercresseyi* (non-infested fish) (n=15 fish per each farm). The *C. rogercresseyi* identified for each of the sampled fish were registered according to their developmental stages established in the Caligus Surveillance Guide following the current Chilean regulations in force (Exempt Resolution No. 060/2022 of the National Fisheries and Aquaculture Service (Sernapesca); Ministry of Economy, Development and Tourism, Government of Chile) [32]. The registered developmental stages of *C. rogercresseyi* correspond to juvenile (chalimus attached to the fish with a rostral filament, approximately 0.8 to 4.2 mm in size), mobile adult (adult stages of females (excluding ovigerous females) and males, with a size of 4.2 mm and the capability of moving freely on the fish), and ovigerous females (adult females of Caligus with ovigerous sacs in the caudal part, also with the ability to move). The total caligus burden in Farm-A and Farm-S was determined by random counting of n=10 fish in four different cages, using macroscopic observation and touch. The presence of caligus in the sampled fish was detailed on a record sheet. The *C. rogercresseyi* infestation load value reported corresponds to the mean number of *C. rogercresseyi* for each caligus stage in the total number of randomly assessed fish. Then, fish classified as infested or non-infested were immediately placed separately in independent tanks (200 L) with constant aeration. Then, fish were sacrificed by tricaine overdose (Sigma-Aldrich; St Louis, MO, USA). Subsequently, a healthy skin tissue sample (1 cm^2^ as described elsewhere [33]) located below the dorsal fin and above the midline was collected, with the absence of any alteration, including skin damage. The samples were carefully pre-processed to rule out the presence of muscle. The samples were then individually placed in a 1.8 mL cryotube with an external thread with an O-ring (SIIbio; Lodi, CA, USA) containing 1 mL of cold absolute ethanol (Sigma; Steinheim, Germany). The samples were immediately frozen at −20 °C, preserving their cold chain until their arrival at the Aquaculture Biotechnology Center, Universidad de Santiago de Chile (Santiago, Chile). Immediately afterwards, the ethanol was discarded with a vacuum pump, and the samples were stored at −80 °C until further processing.

### 2.3. RNA Extraction

The skin sample (approximately 100 mg of tissue) was homogenized in 1 mL of TRIsureTM (Bioline, London, UK) using a tissue master cell disruptor (Omni, Kennesaw, GA, USA) according to the manufacturer’s protocol. The extracted total RNA was resuspended in 30 μL of pyrogen-free miliQ-DEPC water (Invitrogen, Life Technologies, Grand Island, NY, USA). RNA integrity and total RNA concentration were quantified using the Invitrogen Qubit 4 and RNA Quantitation and Qualification kit (Thermo Fisher, Eugene, OR, USA), following the manufacturer’s instructions. Samples included in subsequent analyses had a RIN value ≥ 7 and concentrations greater than 100 ng/μL. Total RNA samples were immediately stored at −80 °C until use.

### 2.4. RNA Sequencing and Analysis

Transcriptomic analysis (RNA-Seq) of skin involved a pooling sampling strategy for the infested (*n* = 3 pools per condition; *n* = 5 different fish per pool; $n_{total}$ = 15 fish analyzed per condition) and non-infested (*n* = 3 groups per condition; *n* = 5 fish per group; $n_{total}$ = 15 fish analyzed per condition) groups, both for samples obtained in autumn and spring.

RNA-seq was performed under an outsourced service contract (Macrogen Inc.; Seoul, Republic of Korea). The amount of RNA was quantified by the Quant-it™ RiboGreen RNA Assay method using the Victor Nivo multimodal reader (PerkinElmer, Shelton, CT, USA) with the Quant-iT RiboGreen RNA Assay kit (Invitrogen, cat.# R11490). RNA condition assessment was performed using the 2100 Bioanalyzer analyzer (Agilent Technologies, Santa Clara, CA, USA), ensuring total integrity of total RNA with a RIN value ≥ 7 prior to processing.

Library preparation was carried out using the TruSeq Gold single-stranded total RNA kit, which was prepared by random fragmentation of cDNA samples, followed by 3^′^ and 5^′^ adapter ligation. Fragments that ligated to the adapter were then amplified by PCR. In the library quality control step, the size of PCR-amplified fragments was verified by running an Agilent Technologies 2100 Bioanalyzer with a DNA 1000 chip. Accurate quantification of library templates was implemented using qPCR following the Illumina quantification protocol for Illumina libraries. Sequencing was performed using Illumina Novaseq 6000 technology, and raw data were obtained through sequencing control software and real-time base calling during the sequencing process on the Illumina sequencer. Subsequently, to convert these files into more accessible, analysis-ready data, the bcl2fastq v2.20 Conversion Software (Illumina, San Diego, CA, USA) was used. This conversion process transforms the data into FASTQ files, which contain the nucleotide sequences, associated quality, and other relevant information for each individual read.

For processing of the raw data (reads), quality control of the raw reads was performed using FastQC version 0.11.9, which generates reports with the quality scores, base composition, k-mer, GC and N contents, sequence duplication levels and overrepresented sequences. Then, alignment of the raw reads to the S. salar reference genomic sequence (Ssal_v3.1 version, NCBI) was performed using STAR software version 2.7.11a [34]. Once alignment was obtained, the mapped reads were assembled and merged into transcripts using the subread package version 2.0.6 [35]. Transcripts per million (TPM) values were used as the gene expression value and differential expression data were normalized using the DESeq2 package in R version 4.3.3 [36] adjusted to the corrected *p*-value (adjusted FDR) < 0.05. Principal Component Analysis (PCA) and hierarchical clustering were also conducted to assess sample variability and replicate consistency. The raw data is available in the Gene Expression Omnibus (GEO) repository with the accession number GSE294058.

### 2.5. Analysis of Differentially Expressed Genes (DEGs) and Interpretation by Functional Networks

DEGs obtained by RNA-seq in the autumn and spring conditions were analyzed using the STRING database version 12.0, https://string-db.org, (accessed on 30 March 2025) to visualize the interactions between the set of DEGs and the biological processes that are part of such interactions. On the other hand, an analysis by ClueGO version 2.5.10 and CluePedia version 1.5.10 [37] was carried out using Cytoscape software version 3.10.1 [38] to determine the biological processes in which the DEGs are involved for each of the experimental conditions. The complete map of interactions that can occur in a living organism was evaluated in order to maximize the information obtained.

### 2.6. DNAse Treatment and cDNA Synthesis

Total RNA samples from Atlantic salmon skin (1 μg) were subjected to DNAse treatment using the PROMEGA Kit (Promega; Madison, WI, USA), according to the manufacturer’s instructions. Following this treatment, cDNA synthesis was performed using the “High Capacity cDNA Reverse Transcription” kit (Applied Biosystems^TM^; Waltham, MA, USA) following the manufacturer’s instructions. Finally, the samples were stored at −20 °C until analysis.

### 2.7. Primer Design and qPCR

To validate the results obtained by RNA-seq, a group of DEGs was selected for autumn and spring according to their participation in key processes such as the immune response (*cebpb*, *c7*, *cd74*), inflammation (*fosl1*, *fosl2*, *jun*, *trib3*), stress (*dusp1*, *hsp90*), and tissue repair (*col2a1*, *tnc*, *cilp*). Primer design was carried out using the Primer-Blast web platform (NCBI). The possible secondary structures of each primer, as well as their specificity, were tested with OligoAnalyzer (version 3.1) and Primer-Blast, respectively. Real-time qPCR reactions were performed using the commercial SYBR Green Master Mix kit (Bio-Rad Laboratories; Hercules, CA, USA) and the CFX Opus 384 Real-Time PCR System thermal cycler (Bio-Rad; USA) following the manufacturer’s instructions. The efficiency of the primers was calculated according to Pfaffl [39] and represented for each gene in Table 1. The qPCR reactions were performed in triplicate in a final volume of 5 μL containing 1 μL of cDNA (5 ng), 0.1 μL of forward (10 mM) and 0.1 μL of reverse (10 mM) primers, 2.5 μL of iTaq, and 1.3 μL of nuclease-free water. The qPCR thermal conditions were 95 °C for 30 s (Hot-Start PCR step) followed by 40 cycles at 95 °C for 30 s and 60 °C for 30 s. Then, a temperature ramping step increasing gradually from 65 °C to 95 C (0.5 °C every 5 s) was included to produce the melting curves in order to verify the amplification of a unique single product in each qPCR reaction. The resulting curves of each qPCR reaction were visualized using Bio-Rad CFX Maestro version 1.1 software.

Several candidates for reference genes (including β*-actin*, the *40S ribosomal protein (fau)*, the *18S ribosomal RNA (18S)*, and the *elongation factor 1α (ef1α)*) were tested using the BestKeeper software to elucidate the one that had less variation between all the samples analyzed. Thus, β*-actin* (for autumn) and *fau* (for spring) were selected as the most idoneous reference genes for analysis. The normalized relative expression for each gene modulated in the infested phenotype was quantified as described by Pfaffl [39] corrected by each primer efficiency using a specific reference gene for each season (β*-actin* for autumn; *fau* for spring) and normalized to the control group. The normalized relative expression values were transformed to log2 and plotted in GraphPad version 9.

### 2.8. Statistical Analysis

To identify differentially expressed genes (DEGs), massive RNA sequencing data were processed using the DESeq2 package in Rstudio. An initial filtering step was implemented to exclude genes with less than 10 total reads, focusing the analysis on genes with significant expression levels.

Differential expression analysis was performed using a negative binomial regression model to contrast “Infested” versus “Non Infested” conditions. DEGs were identified through an adjusted *p*-value (FDR) less than 0.05, indicating statistical significance after correction for multiple testing. Volcano plots were generated to visualize the magnitude and significance of expression changes, highlighting genes with log changes (|log2Fold Change| > 1). In addition, Principal Component Analysis (PCA) of the DEGs was performed to examine the dispersion and separation between samples, allowing the identification of differential expression patterns between experimental conditions.

Functional network analysis was performed using STRING where functional enrichment analysis of protein–protein interaction (PPI) networks was performed for all DEGs with a confidence score 0.4 using the *S. salar* organism as a model. In addition, ClueGO, a Cytoscape plugin that integrates gene ontology and metabolic pathway annotations, was used to provide biological context for the identified DEGs. ClueGO was configured to perform a functional analysis by selecting the “Functional Analysis” mode and setting the network specificity to medium. An enrichment/depletion test using a bilateral hypergeometric test, with a Bonferroni step-down correction, was used to determine statistically significant functions. Only pathways with a *p*-value less than 0.05 were included, using the option “Show only Pathways with pV ≤ 0.05” to ensure that only significantly enriched pathways were considered. In addition, the “Use GO Term Fusion” option was enabled to combine redundant GO terms and simplify the interpretation of the results.

For RNA-seq validation by qPCR, “Graphpad Prism 9” software was used to evaluate possible significant differences of DEGs in the autumn and spring seasons. Two-way ANOVA with Šidák’s multiple comparisons post-test was performed to determine if significant differences existed between qPCR and RNA-seq gene expression (*p*-value < 0.05).

## 3. Results

### 3.1. Infestation Profile and Life Stage of C. rogercresseyi Across Seasons

**Figure 1:**
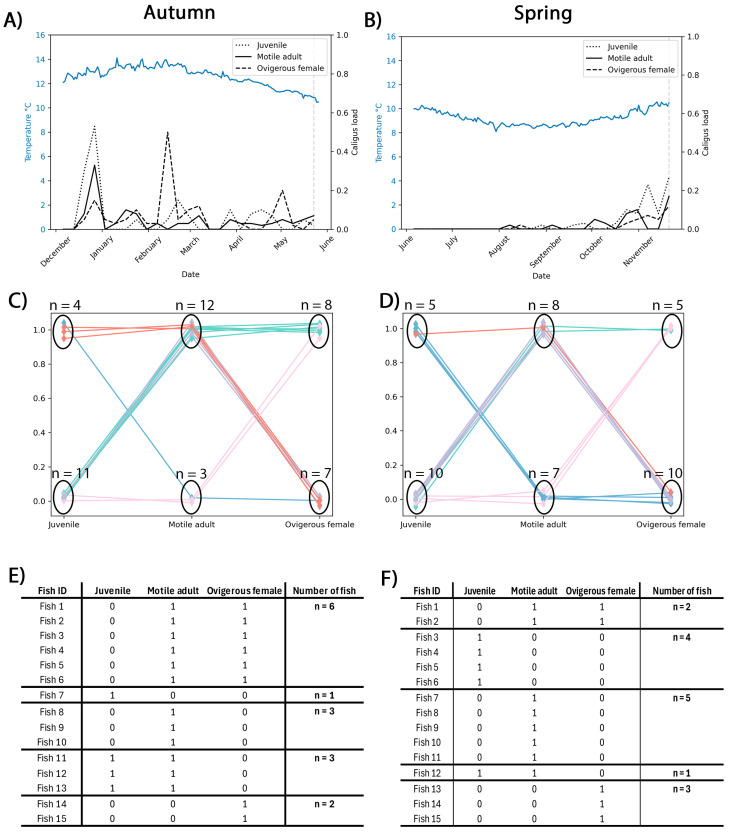
Temperature and *Caligus rogercresseyi* load profile in autumn (Farm-A) and spring (Farm-S) for the fish farms included in the study. (**A**,**B**) Mean seawater temperature (blue line) and mean *C. rogercresseyi* infestation load for their three developmental stages were determined for each individual, including juvenile (dotted line), motile adult (solid line), and ovigerous female (dashed line) during (**A**) autumn and (**B**) spring. (**C**,**D**) Representation by parallel coordinate plots of the *C. rogercresseyi* infestation load in (**C**) autumn and (**D**) spring for each fish included in the transcriptomic analysis. Each line represents one single fish (*n* = 15 infested fish per season). The different-color lines indicate the infestation pattern for each single fish based on the registered *C. rogercresseyi* developmental stages. (**E**,**F**) Details of the load and growth stages of *C. rogercresseyi* for the (**E**) autumn and (**F**) spring fish included in the study. For each fish (Fish ID), the number of ectoparasites identified at the juvenile, motile adult, and ovigerous female stages is represented.

During autumn (Figure 1A), an increase in *C. rogercresseyi* infestation load was observed in both juvenile and motile adult stages between December 2021 and March 2022, coinciding with a progressive steady rise in seawater temperature that peaked at 14 °C in January and February. Ovigerous females exhibited sporadic and less pronounced peaks compared with the other developmental stages. On the other hand, spring showed an infestation load that remained low from June to September and progressively increased toward November, likely due to the seasonal rise in water temperature from winter to spring (Figure 1B). A slight increase in all the *C. rogercresseyi* developmental stages was registered in November 2022, suggesting a seasonally favorable condition for the *C. rogercresseyi* attachment and completion of the ectoparasite life cycle in response to rising temperatures.

The infestation load and developmental stage of *C. rogercresseyi* for each fish farm included in the study are illustrated in the Sankey diagrams in Figure 1C,D. In autumn, Farm-A was observed to show a marked presence of *C. rogercresseyi*, mainly at the motile adult and ovigerous female developmental stages (Figure 1C). In contrast, in spring, Farm-S recorded a slight increase in the number of fish with *C. rogercresseyi* at the juvenile stage compared to that observed for fish sampled in autumn (Figure 1D). Furthermore, spring showed a higher number of fish with *C. rogercresseyi* at the motile adult stage, along with a lower number at the ovigerous female stage compared to that observed in autumn (Figure 1D).

When analyzing the total infestation load of *C. rogercresseyi* in the fish included in this study, a diversity in the combination of different developmental stages is observed in autumn, with a notable proportion of fish simultaneously hosting juvenile, motile adult, and ovigerous female stages (*n* = 6 fish), indicating higher parasite burden and advanced life cycle progression (Figure 1E). On the other hand, most of the fish sampled in spring harbored either a single developmental stage (*n* = 12 fish), with fewer individuals (*n* = 3) carrying multiple stages concurrently (*n* = 2 fish with one motile adult and one ovigerous female; *n* = 1 fish with one juvenile and one motile adult), reflecting an earlier or less established stage of infestation compared with autumn (Figure 1F).

### 3.2. Differential Transcriptomic Profiling of the Skin of Atlantic Salmon Infested with C. rogercresseyi Under Productive Conditions in Autumn and Spring

**Figure 2:**
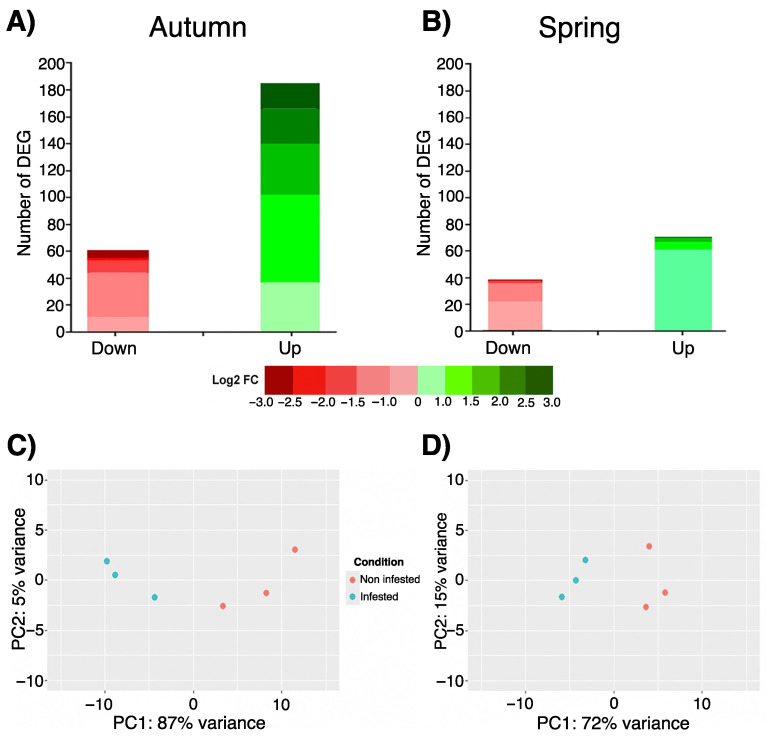
Transcriptomic analysis based on RNA-seq of Atlantic salmon skin infested with *C. rogercresseyi* under productive conditions in autumn and spring. The total number of differentially expressed genes (DEGs) for (**A**) autumn and (**B**) spring. The range of gene expression magnitude (represented as log2 fold change) is shown in red (downregulated DEGs) and green (upregulated DEGs). Principal Component Analysis (PCA) for (**C**) autumn and (**D**) spring. In both PCAs, the spheres correspond to each of the pools analyzed (*n* = 3 pools per season and condition, made up of *n* = 5 different fish in each pool) (infested, in blue; non-infested, in red).

In this study, differential gene expression was determined in autumn and spring skin of Atlantic salmon infested with *C. rogercresseyi* under productive conditions. Transcriptomic analysis of the skin infested in autumn yielded 252 differentially expressed genes (DEGs). Among them, 185 DEGs were upregulated; specifically, 37 genes showed a modulation between 0 < log2FC < 1.0, 65 DEGs a modulation between 1.0 < log2FC < 1.5, 38 DEGs a modulation between 1.5 < log2FC < 2.0, 26 DEGs a modulation between 2.0 < log2FC < 2.5, and 19 DEGs registered a modulation log2FC > 3.0 (Figure 2A). On the other hand, a total of 67 DEGs were found to be downregulated, 13 DEGs with a modulation between −1.0 < log2FC < 0, 36 DEGs between −1.5 < log2FC < −1.0, 10 DEGs with a modulation between −2.0 < log2FC < −1.5, 2 DEGs with a modulation between −2.5 < log2FC < −2.0, and 6 DEGs with a modulation log2FC < −3.0 (Figure 2A).

The transcriptomic analysis of the skin infested in spring showed 103 DEGs, where 70 DEGs were upregulated, 61 DEGs showed a modulation between 0 < log2FC < 1.0, 6 DEGs with a modulation between 1.0 < log2FC < 1.5, and 3 DEGs were modulated with a log2FC > 1.5. In addition, 33 DEGs were downregulated in spring, where 19 DEGs modulated between −1.0 < log2FC < 0, 12 DEGs presented modulation between −1.5 < log2FC < −1.0, and 2 DEGs modulated with log2FC < −1.5 (Figure 2B).

Principal Component Analysis (PCA) was also conducted to examine the variability among the samples according to the transcriptional profile of Atlantic salmon skin. The PCA revealed clustering between the samples of each experimental group (infested and non-infested), as well as a clear segregation between the samples of infested fish compared to non-infested fish in terms of transcriptional profile, both in autumn (Figure 2C) and spring (Figure 2D).

**Figure 3:**
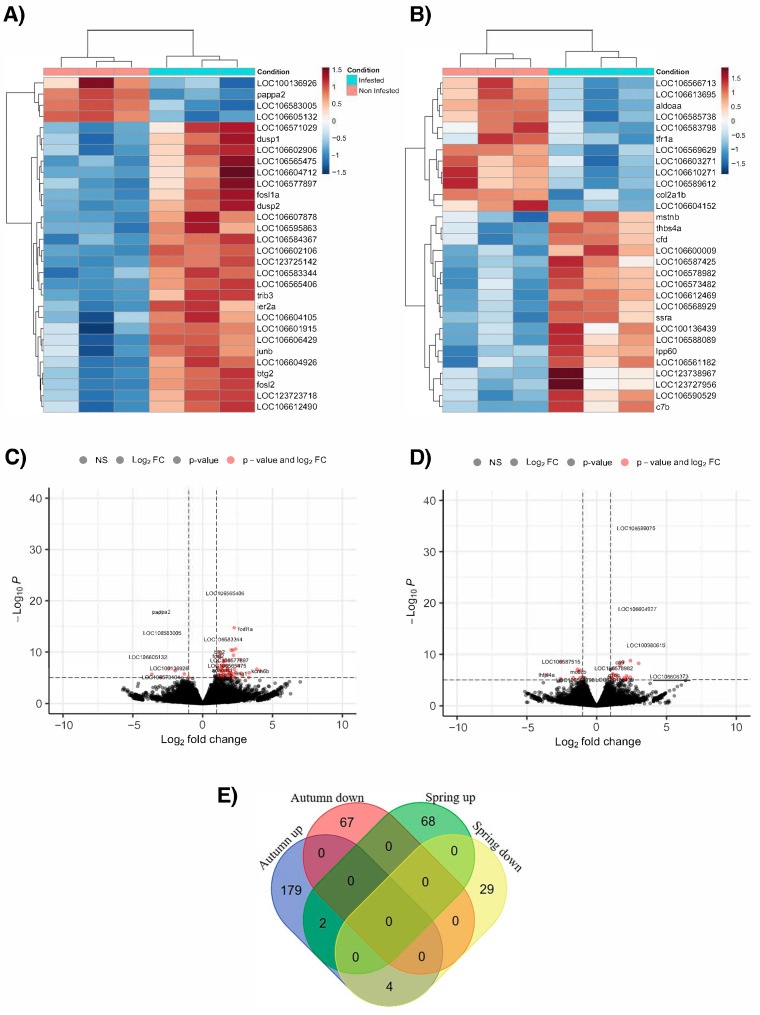
Transcriptomic analysis based on RNA-seq of Atlantic salmon skin infested with *C. rogercresseyi* under productive conditions in autumn and spring. (**A**,**B**) Hierarchical heatmap for (**A**) autumn and (**B**) spring. In both hierarchical maps, the expression patterns of the top 30 genes in each season are shown. The color gradient line indicates upregulation (red) and downregulation (blue). Each column corresponds to each of the pools analyzed by condition (infested, in light blue; non-infested, in red) in each evaluated season. (**C**,**D**) Volcano plot for (**C**) autumn and (**D**) spring. In both plots, the DEGs considered statistically significant (red spots) are represented according to their expression value (Log2 fold change > 1) and a *p*-value < 0.05. (**E**) Venn diagram representing the exclusive and shared DEGs according to their modulation (up- or downregulated) in infested fish compared to non-infested fish in autumn and spring.

The hierarchical heatmaps confirm the segregation observed in the PCA, showing that in both autumn and spring, both phenotypes form independent clusters based on the expression of the top 30 DEGs. Moreover, both hierarchical heatmaps are in line with what is observed in Figure 2A,B. In fact, in autumn, the infested phenotype shows significant upregulation of genes and a lower number of downregulated DEGs (Figure 3A). On the other hand, in spring, gene expression is more balanced, although with a higher number of upregulated than downregulated DEGs (Figure 3B). In the volcano plot, key genes with significant changes in expression Log2 fold change |1| and a *p*-value < 0.05 stand out (Figure 3C,D). The Venn diagram shows that DEG modulation in the infested phenotype is highly conditioned by seasonality. This is because the vast majority of the DEGs are exclusively upregulated/downregulated. Among them, the transcriptional profile showed 179 upregulated and 67 downregulated DEGs for autumn, while in spring, 68 upregulated and 29 downregulated DEGs were recorded, as shown in Figure 3E. The complete list of differentially expressed genes in both autumn and winter, as well as their modulation (down/upregulated), is presented in Supplementary Table S1.

### 3.3. Functional Network Analysis

**Figure 4:**
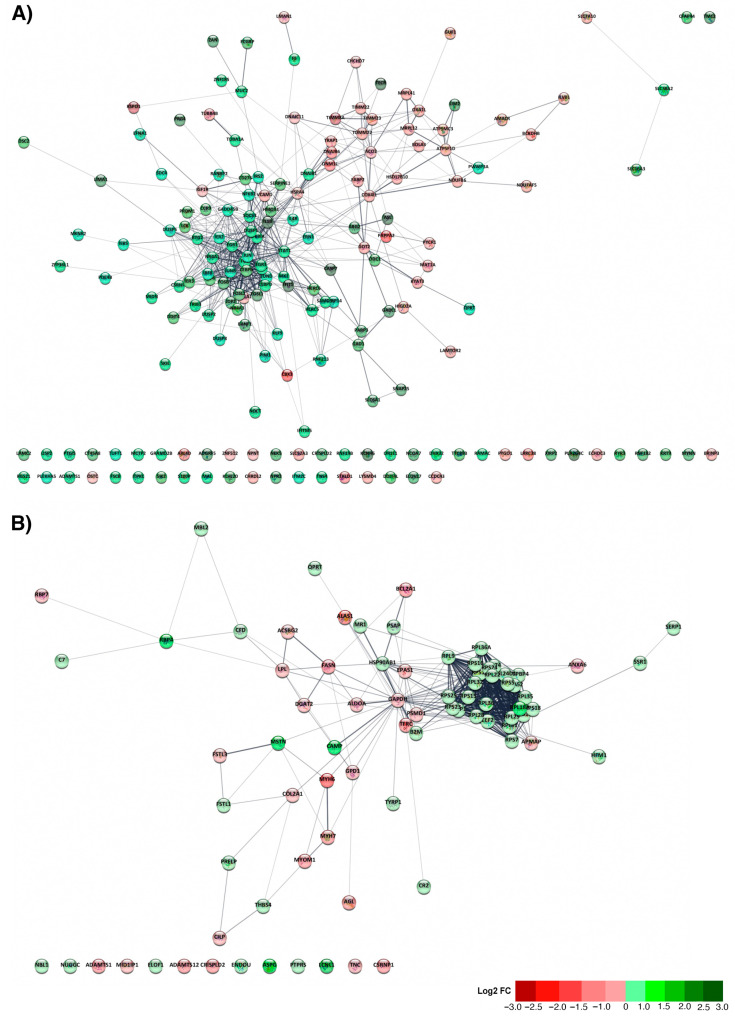
Functional network analysis for differentially expressed genes (DEGs) in the skin of Atlantic salmon infested with *C. rogercresseyi* under productive conditions in autumn and spring. (**A**) Functional network obtained for the DEGs found in autumn (181 nodes; 593 edges). (**B**) Functional network obtained for the DEGs found in spring (80 nodes; 463 edges). Each node represents a DEG obtained from the transcriptomic analysis. The modulation profile of each DEG is represented in a red (downregulated) and green (upregulated) gradient according to its magnitude of change (Log2 fold change). The connections between nodes indicate significant functional interactions identified using the STRING database.

Based on the total DEGs obtained from the transcriptomic analysis, a functional network analysis was performed to determine the interaction between each gene in the skin of Atlantic salmon infested with *C. rogercresseyi* in autumn and spring. In autumn, 181 out of 252 DEGs (equivalent to 71.54%) showed a network with 593 interactions (edges), leading to multiple interactions (Figure 4A). In spring (Figure 4B), 80 out of the 103 DEGs found (equivalent to 77.67%) were part of the functional network, which stands out for its 436 interactions that are responsible for the formation of a highly connected central core.

**Figure 5:**
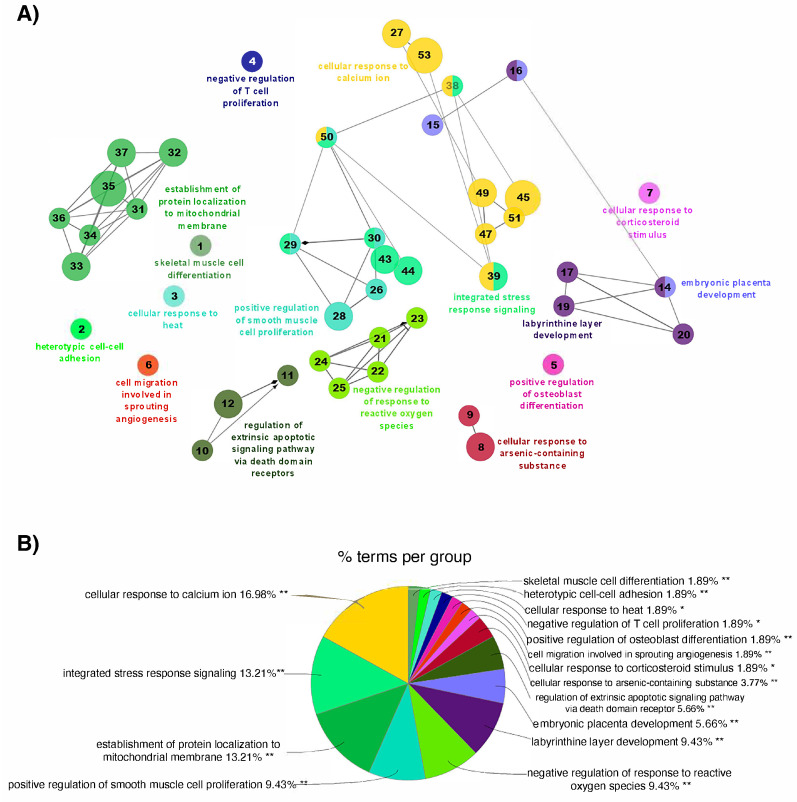
Functional network for the biological processes enriched from the transcriptome of Atlantic salmon skin infested with *C. rogercresseyi* under productive conditions in autumn. (**A**) Functional network for the enriched biological processes obtained from the DEGs in the skin of Atlantic salmon infested with *C. rogercresseyi* compared to non-infested fish. Each node represents a significantly enriched GO (gene ontology) term (45 nodes, 46 term connections). The size of each node indicates the number of DEGs involved in the process. The numbers in each node represent the associated process, which is detailed in Supplementary Table S2. The connections indicate functional relationships between the terms, and the colors represent groups of related biological processes. (**B**) Distribution of the enriched GO terms. The pie chart shows the enriched GO terms indicated in (**A**) according to their percentage (%) representation based on the total DEGs in *C. rogercresseyi*-infested fish in autumn. The level of significance of the terms is shown as ** (*p* value < 0.001), * (0.001 < *p* value < 0.05).

Subsequently, the ClueGO plugin in Cytoscape was used to determine the biological context of response among the identified DEGs. The functional network shows, for autumn, the interaction between cellular response to calcium ion, integrated stress response signaling, and processes related to cell development and proliferation, and positive regulation of smooth muscle cell proliferation. Linked with the stress response, the interactome also showed the representation of the cellular response to corticosteroid stimulus, negative regulation of response to reactive oxygen species (ROS), and the cellular response to heat. At the cellular level, we also registered processes related to cell–cell adhesion, angiogenesis, and osteoblast differentiation. At the defensive level, the skin showed a transcriptome associated with the apoptotic signaling pathway and the negative regulation of T-cell proliferation (Figure 5A). On the other hand, the most enriched terms included “cellular response to calcium ion” (16.98%), “integrated stress response signaling” (13.21%), “protein localization to mitochondrial membrane” (13.21%), “positive regulation of smooth muscle cell proliferation” (9.43%), and “negative regulation of T cell proliferation” (1.89%) (Figure 5B). The complete list of the enriched biological processes obtained in autumn, as well as the genes involved, is presented in Supplementary Table S2.

**Figure 6:**
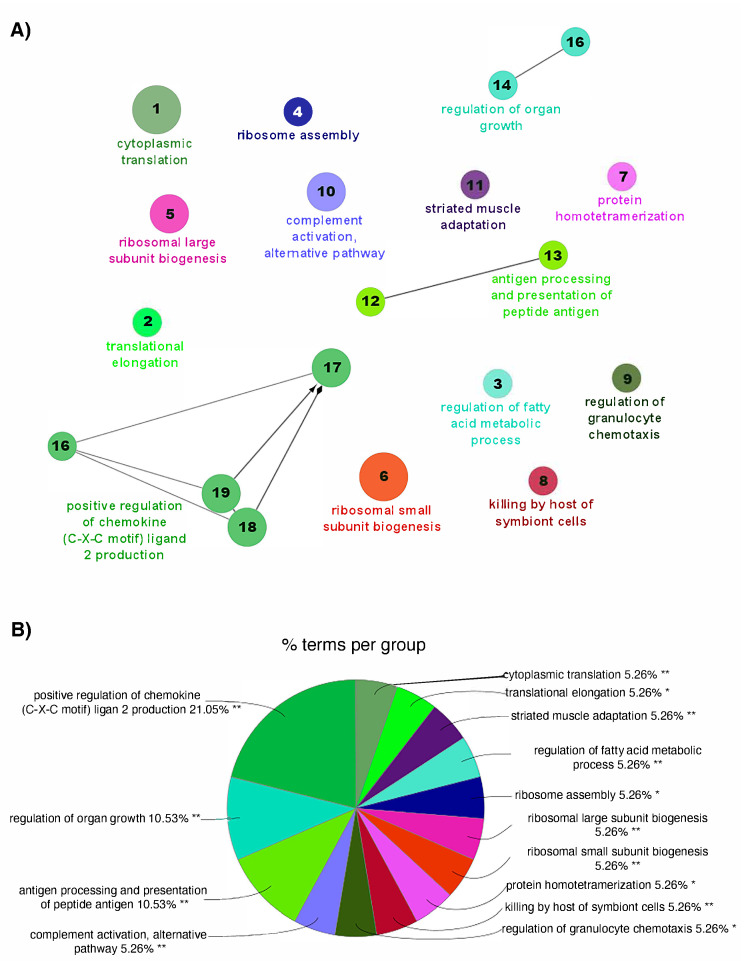
Functional networks for the biological processes enriched from the transcriptome of Atlantic salmon skin infested with *C. rogercresseyi* under productive conditions in spring. (**A**) Functional network for the enriched biological processes obtained from the differentially expressed genes (DEGs) in the skin of Atlantic salmon infested with *C. rogercresseyi* compared to non-infested fish. Each node represents a significantly enriched GO (gene ontology) term (19 nodes, 6 terms connections). The size of each node indicates the number of DEGs involved in the process. The numbers in each node represent the associated process, which is detailed in Supplementary Table S2. The connections indicate functional relationships between the terms, and the colors represent groups of related biological processes. (**B**) Distribution of the enriched GO terms in spring. The pie chart shows the enriched GO terms indicated in (**A**) according to their percentage (%) representation based on the total DEGs in *C. rogercresseyi*-infested fish in spring. The level of significance of the terms is shown as ** (*p* value < 0.001), * (0.001 < *p* value < 0.05).

In spring, no interconnected biological processes were observed to form an interactome. However, the enrichment analysis reveals a series of biological processes. Among them, the presence of multiple processes associated with biogenesis stands out (translational- and ribosome-related processes, protein homotetramerization). Processes associated with muscular adaptation and growth regulation were also identified. Additionally, multiple processes related to the immune response were observed, including complement activation, positive regulation of chemokine (C-X-C motif) ligand 2 production, regulation of granulocyte chemotaxis, and antigen processing and presentation of peptide antigen (Figure 6A). The enrichment showed that the most representative processes include “positive regulation of chemokine (C-X-C motif) ligand 2” (21.05%), “antigen processing and presentation of peptide antigen” (10.53%), and “regulation of organ growth” (10.53%) (Figure 6B). The complete list of the enriched biological processes obtained in spring, as well as the genes involved, is presented in Supplementary Table S3.

### 3.4. Validation of RNA-seq by Real-Time qPCR

**Figure 7:**
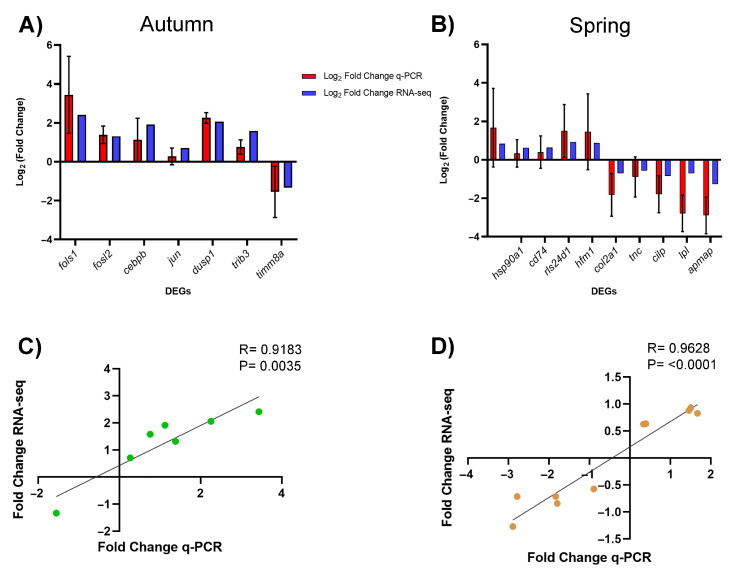
RNA-seq validation by qPCR in differentially expressed genes in the skin of Atlantic salmon infested with *C. rogercresseyi* under productive conditions. (**A**) Comparison of gene expression between RNA-seq and qPCR, represented as Log 2 fold change for autumn. (**B**) Comparison of gene expression between RNA-seq and qPCR, represented as Log 2 fold change for spring. The bars represent the mean ± standard error of expression obtained by qPCR for each pool previously analyzed by RNA-Seq (*n* = 3 pools per season and by condition, made up of *n* = 5 different fish in each pool). Statistical analysis (two-way ANOVA with Šidák’s multiple comparisons (*p*-value < 0.05)) shows no statistical differences in all DEGs evaluated using both transcriptomic strategies. (**C**) Pearson correlation for autumn (**C**) (R = 0.9183, *p* = 0.0035). (**D**) Pearson correlation for spring (R = 0.9628, *p* < 0.0001). In both graphs (**C**,**D**), the relationship between the two gene expression analysis strategies is represented (RNA-seq and qPCR), reinforcing the consistency of the results from both methodologies. *fosl1: FOS-like 1, AP-1 transcription factor subunit a*; *Fosl2: fos-related antigen 2*; *cebpb: CCAAT enhancer binding protein beta*; *jun: Jun proto-oncogene, AP-1 transcription factor subunit*; *dusp1: dual specificity phosphatase 1*; *trib3: tribbles pseudokinase 3*; *timm8a: translocase of inner mitochondrial membrane 8 homolog a*; *c7: complement component 7b*; *hsp90ab1: heat shock protein 90, alpha (cytosolic), class B member 1*; *cd74: CD74 molecule, major histocompatibility complex, class II invariant chain a*; *rsl24d1: probable ribosome biogenesis protein RLP24*; *hfm1: nucleolar RNA helicase 2*; *col2a1: collagen, type II, alpha 1b*; *tnc: tenascin Ca*; *clip: cartilage intermediate layer protein, nucleotide pyrophosphohydrolase*; *lpl: lipoprotein lipase*; *apmap: adipocyte plasma membrane associated protein*.

To validate the results obtained through RNA-seq, qPCR was performed on differentially expressed genes selected in autumn and spring. The comparison between RNA-seq and qPCR expression values showed similar expression levels, reflected in no statistical differences for all DEGs evaluated using both transcriptomic strategies. Additionally, a correlation between both seasons was observed, evaluated through Pearson correlation analysis for both autumn (R = 0.9183, *p* = 0.0035) and spring (R = 0.9628, *p* < 0.0001) (Figure 7).

## 4. Discussion

In the present study, we evaluated the impact of seasonal variation on gene expression in the skin of Atlantic salmon under conditions of natural infestation by *C. rogercresseyi*. Throughout the year, water temperature is subject to natural fluctuations associated with annual seasonality during the growth of Atlantic salmon in marine farms. As expected, historical health records (2008–2024) from Atlantic salmon farming centers in southern Chile indicate that the highest maximum temperatures occur in summer, while the lowest surface water temperatures are recorded in winter. Similarly, comparable temperatures at the water surface occur in autumn (descending temperature ramp, from summer to winter) as in spring (ascending temperature ramp, from winter to summer). In this regard, it is interesting to note that historical health reports of *C. rogercresseyi* infestation in Chile show that the peak of annual infestation has mostly occurred in autumn in fifteen of the last sixteen years [40,41,42,43,44,45,46,47,48,49,50,51]. Similar observations were made on the west coast of Scotland, where the infestation load of *Lepeophtheirus salmonis* in Atlantic salmon was studied. It was recorded that the highest infestation load occurs during the summer-to-winter transition, i.e., during the descending temperature ramp (autumn) [52]. These findings contrast with reports stating that the sea louse completes the different phases of its cycle more quickly at higher temperatures compared to lower temperatures [9,53,54]. This apparent discrepancy may be due to the lack of studies aimed at characterizing the response mechanisms of Atlantic salmon skin infested with *C. rogercresseyi* during different seasons throughout the year. Consequently, the poikilothermic nature of fish and their ability to respond differentially based on seasonal fluctuations in annual water temperature ramps could be responsible for the majority of the annual infestation peaks, which occur predominantly in autumn in the Aysén Region. For this reason, this study focused on evaluating the transcriptomic response of the skin of Atlantic salmon infested with *C. rogercresseyi* in autumn (descending temperature ramp) and spring (ascending temperature ramp) to shed light on the response mechanisms based on seasonality in response to sea louse infestation under productive conditions. Our results show a clear impact of seasonality on the transcriptomic response of the skin of Atlantic salmon in response to natural infestation by *C. rogercresseyi* in marine farming conditions. The clear effect of seasonality on the differential expression profile is reflected in the fact that only 6 DEGs out of the 349 total DEGs identified are shared between any of the seasons, while the other 343 DEGs are exclusive to one of the seasons (autumn; spring), and differentially modulated (upregulated or downregulated). This marked differential expression profile highlights the relevance of conducting studies under natural productive conditions that consider the poikilothermic nature of fish and, along with this, their modulatory capacity to respond to fluctuating environmental conditions throughout the year in which they are raised.

The effect of seasonality on the immune response is critically underexplored in fish. To the best of our knowledge, there are no reports for mucosal tissues (including skin mucosa) in this matter. Moreover, one of the gaps in this area of knowledge is the need to develop research focused on studying the variation in the immune response of Atlantic salmon throughout the different seasons of the year in a productive environment. In this way, our study stands out as pioneering in describing the mucosal response of the skin in Atlantic salmon to *C. rogercresseyi* under open-sea cage conditions in marine farms. In our study, the results show a differential expression profile in various processes associated with the immune response in autumn and spring. In autumn, our data recorded the negative regulation of T-cell proliferation in Atlantic salmon infested with *C. rogercresseyi*. Among the identified genes, our study found the upregulation of *syndecan 4*, which is considered a highly potent inhibitor of T-cell activation [55]. In this way, the lack of T-cell proliferation would have implications for the execution of defense response mechanisms in Atlantic salmon against the ectoparasite. In a previous study conducted under controlled laboratory conditions, Atlantic salmon infested with *C. rogercresseyi* exhibited a specific immune response characterized by a Th1 response [5], a key component of the cell-mediated immune response and the resolution of intracellular infections. In mammals, it has been described that a key process in the response to extracellular parasites is the activation of adaptive immunity mediated by T cells, aimed at activating a response in a T-helper context. However, in Atlantic salmon, the research focus has been widely on *L. salmonis* (one of the sea lice species identified in the North Hemisphere) rather than *C. rogercresseyi*. These copepods parasitize salmon during the marine phase of their life cycle by attaching to their skin or fins and feeding from their blood and tissue [10]. In teleosts resistant to sea lice, such as Coho salmon (*Oncorhynchus kisutch*), it has been described that their immune response to *L. salmonis* includes T-cell proliferation [56]. In contrast, Atlantic salmon is a species susceptible to infestation by *C. rogercresseyi* [10]. This background, along with the upregulation of IL-4 registered only in Coho but not in Atlantic salmon, suggests that the response mediated by T-helper type 2 (Th2) cells would play a key role in the defense mechanism against the ectoparasite [30,56]. In our results, the lack of T-cell proliferation would cause truncation in the activation of the T-cell-mediated response by compromising the activation of immunity in a Th2 context, which seems to be key in resistance to sea louse infestation. Hence, the finding about the negative regulation of T-cell proliferation in Atlantic salmon infested with *C. rogercresseyi* could be one of the reasons that explains why the peak of annual infestation by *C. rogercresseyi* takes place in autumn in the Aysén Region (Chile). By contrast, our study recorded the modulation of genes associated with antigen processing and presentation of peptide antigens in spring. Among the genes, we found the upregulation of *beta-2 microglobulin (b2m)*, a light chain component of Major Histocompatibility Complex (MHC) class I molecules associated with the activation of the CD8+ T cell-mediated immune response [57]; *cd74*, a transmembrane glycoprotein primarily involved in the assembly and trafficking of MHC class II molecules [58] that are responsible of the exogenous peptide antigen presentation to CD4+ T-helper cells; and *mr1 (MHC Class I-Related Protein 1)*, a highly conserved, non-polymorphic MHC class I-like molecule that presents microbial metabolites to a specialized subset of T cells called mucosal-associated invariant T (MAIT) cells [59]. Although we did not find modulation of genes associated with T-cell activation in our study during spring, the upregulation of molecules involved in their activation suggests that this process may be carried out in a mucosal-specific context.

The Th2 immune response can limit tissue injury and maintain tissue homeostasis [60,61]. It is known that sea lice cause damage and activate the expression of genes associated with tissue repair. In our study, we did not observe modulation of genes involved in the Th2 response, which is also associated with tissue repair. Instead, we registered the upregulation of *arginase 2* and *CCAAT/enhancer-binding protein beta* in autumn, two molecules that show a connected role in macrophage polarization, specifically in the induction of the M2 phenotype [62,63], which is associated with immunomodulation [64], wound healing [63], and tissue repair [64]. Interestingly, these genes are also part of the enrichment associated with the negative regulation of T-cell proliferation. Thus, these genes may be involved in regulating T-cell proliferation and promoting tissue repair mechanisms that could be associated with enhancing the immune response in an M2 context. In our study, we also observed during autumn the upregulation of *klf4* and *egr3*. The transcription factor *klf4* plays a key role in the regulation of tissue repair through the maintenance of endothelial barrier integrity, and it regulates the expression of *VE-cadherin*, a key protein of adherens junctions in endothelial cells [65]. On the other hand, *egr3* participates in tissue repair by promoting the expression of profibrotic genes in fibroblasts in response to *tgf-β*, such as *col1a1*. Its expression increases in injured tissue and in fibrosis models, indicating that *egr3* regulates the activation of myofibroblasts during tissue remodeling [66]. Simultaneously, cell migration involved in sprouting angiogenesis was also observed in our study. The upregulation of all the genes associated with this process, including the *early growth response protein 3 (egr3)*, *heme oxygenase-1 (hmox1)*, *kruppel-like factor 4 (klf4)*, and the *nuclear receptor subfamily 4 group A member 1 (nr4A1)*, indicates endothelial cell migration, modulation of growth factor signaling pathways (like VEGF), and angiogenesis [67,68,69,70]. The identification of heterotypic cell–cell adhesion in our study (i.e., the attachment of a cell to a cell of a different type via adhesion molecules) reinforces the idea that tissue repair mechanisms take place in the Atlantic salmon skin infested with *C. rogercresseyi* in autumn.

The modulation of these processes, which are upregulated during autumn in the context of *C. rogercresseyi* infestation, suggests that, in addition to activating specific immune responses, the host may be promoting mechanisms to restore tissue integrity and counteract the epithelial damage induced by the ectoparasite.

Studies in this still-incipient area of research suggest that the effect of seasonality on immunity in salmonids is associated with the adaptive strategies of fish responding to different environmental conditions that change throughout the year [71,72]. In line with this idea, the temperature ramp would play a key role in modulating this adaptive strategy. Indeed, a decrease in temperature has been associated with reduced immunity; by contrast, an increase in temperature appears not to significantly affect it [73]. These antecedents reinforce the idea that a decreasing temperature ramp would compromise the immunological capacity of fish, thereby increasing their vulnerability to pathogens.

The decreased immune capacity observed in autumn may be attributed to the activation of the stress response. It is well known that infestation by sea lice induces the promotion of the stress response [74]. Linked to this idea, we identified that infestation with *C. rogercresseyi* favors the cellular response to corticosteroid stimulus in Atlantic salmon skin during autumn, considered a biomarker of systemic stress response activation [75]. Thus, we registered the modulation of genes directly associated with *(Krueppel-like factor 9 (klf9)*, and *Zinc Finger Protein 36*, *C3H1 Type-like 1 (zfp36L1))* and influenced by glucocorticoids *(Insulin-like growth factor 1 receptor (igf1r))*. The activation of biological processes, modulated as a consequence of the stress response, requires the binding of corticosteroids to the glucocorticoid receptor in the cytoplasm [76]. This binding leads to the translocation of GR to the nucleus, where it binds to a specific DNA sequence (promoter and enhancer regions) known as Glucocorticoid Response Elements (GREs). Its action promotes the expression of key genes, including *klf9* and *zfp36L1*, in a cortisol-dependent manner [77,78]. Thus, the upregulation of *klf9* and *zfp36L1* registered in our study is probably the consequence of the activation of the stress response in Atlantic salmon infested with *C. rogercresseyi* in autumn. On the other hand, the activation of the stress response in Atlantic salmon infested with *C. rogercresseyi* in autumn but not in spring could be the result of gene modulation in response to temperature. Indeed, only the transcriptomic profile for autumn registered the cellular response to heat in the Atlantic salmon skin. Thus, the activation of the stress response in autumn can be directly attributed to the seasonal variation in response to the ectoparasite within the context of Atlantic salmon poikilothermy.

The integrated stress response is an evolutionarily conserved intracellular signaling network that helps the cell, tissue, and organism to adapt to a variable environment and maintain health [79]. Unlike the general stress response mediated by hormones like cortisol, the ISR is a cellular response that seeks to protect and adapt the cell to damage that converges on the phosphorylation of the alpha subunit of eukaryotic translation initiation factor 2 *(eIF2α)* [80]. To the best of our knowledge, our study is the first to report evidence of an integrated stress response (ISR) in the skin of Atlantic salmon infested with *C. rogercresseyi*. Importantly, this response also appears to be seasonally conditioned, being present in autumn but not in spring. Thus, our study showed the upregulation of all DEGs associated with ISR at different levels of activation, including its downstream pathway *(activating Transcription Factor 3 (atf3))* [81], basic leucine zipper (bZIP) transcription factors *(CCAAT enhancer binding protein beta (cebpb)* and *delta (cebpd))* [82,83], components of the AP-1 complex whose expression is part of the ISR’s cellular adaptive program *(proto-oncogene c-Fos (fos); Jun proto-oncogene; AP-1 transcription factor subunit (jun); jun B proto-oncogene (junB))* [84,85], and stress-inducible proteins *(dnaJ homolog subfamily B member 1 (dnaJB1); heme oxygenase 1 (hmox1))* [86,87].

Oxidative stress is one of the mechanisms that promotes the activation of the integrated stress response [88,89]. Previous studies reported that the presence of sea lice triggers a cascade of physiological responses in the host fish, including inflammation and immune activation, which can lead to an increase in reactive oxygen species (ROS) and, consequently, oxidative stress [74,90]. In this way, we recorded in our study the modulation of components that are associated with the negative regulation of oxidative stress produced by ROS, including the upregulation of the *nuclear receptor subfamily 4, group A, member 3 (nr4A3)* [91], and the downregulation of *pyrroline-5-carboxylate reductase 1, mitochondrial-like (pycr1)* [92], and *tnf receptor-associated protein 1 (TRAP1)* [93]. Mitochondria and oxidative stress are intimately linked, forming a complex relationship that is crucial for cellular health [94]. Thus, it is not surprising to find several processes associated with mitochondrial functionality in our study. In fact, downregulation of the electron transport chain (ETC) was recorded in *C. rogercresseyi*-infested fish in autumn, which could also be related to the upregulation of the integrated stress response. Indeed, chronic stress has been observed in mammals to affect the ETC, especially complex II, allowing adjustment of metabolism to situations that compromise its health via the mitochondria [95,96]. When the stress is severe or prolonged, the ISR can switch to a pro-apoptotic program, leading to programmed cell death (apoptosis) and the elimination of damaged cells. This idea is probably the reason we also found processes associated with the extrinsic apoptotic signaling pathway via death domain receptors and their regulation. Taken together, we hypothesize that the activation of the ISR is a strategy to restore cellular homeostasis by protecting the skin tissue cells from damage caused by infestation with *C. rogercresseyi*.

On the other hand, the response of the skin against *C. rogercresseyi* in spring showed no modulation of the processes related to the activation of the stress response, either at the physiological level or in its integrated response, as this study identified for autumn. These results suggest that the activation of stress in response to *C. rogercresseyi* does not depend exclusively on ectoparasite infestation but appears to be modulated by seasonality. This antecedent reinforces the importance of considering environmental variability in understanding, from a poikilothermal perspective, the biological processes modulated by biotic threats that impact the health of teleost fish. The lack of activation of the stress response does not, from a physiological perspective, influence the activation of various biological processes, including the immune response. Thus, this improved physiological condition in spring favors the activation of several Atlantic salmon skin mechanisms in response to *C. rogercresseyi* infestation. The immune response exhibited in spring includes the upregulation of processes related to the innate (such as complement pathway activation and granulocyte chemotaxis) and adaptive immunity (antigen processing and antigen presentation). The “complement activation, alternative pathway” is a key component of the innate immune response, playing a crucial role in identifying pathogens and clearing parasites [97,98]. The promotion of the complement response has been previously reported after louse infection (*L. salmonis*) conducted under lab conditions [99]. In our study, this process was represented by the upregulation of the *complement component 7b (c7)*, *complement factor D (cfd)*, and the *complement c3d receptor 2 (cr2)* in response to infestation by *C. rogercresseyi* in spring. Previous evidence has shown that the temperature does not influence the complement response [100,101], although it depends on the fish species [12]. CR2 is a surface glycoprotein whose expression has been reported in epithelial cells [102,103,104] and antigen-presenting cells [105]. In addition, we also identified the upregulation of the *mannose-binding protein C (mbl2)*, a protein that leads to the cleavage of C3 of the complement system and the generation of the fragments C3a, C3b, and C3d [106]. Thus, previous studies in mammals have reported that CR2 binds to cleaved C3 (including C3b and C3d), forming a complex with the antigen that is recognized by the B-cell receptor (BCR) [107].

*The mannose-binding protein C (mbl2)* is also involved in the recognition and binding to specific carbohydrate patterns (like mannose and N-acetylglucosamine) found on the surface of a wide range of microorganisms [108]. Mannose and N-acetylglucosamine are important monosaccharides (simple sugars) that play various roles in the biology of many organisms, including parasites like sea lice. Chitin, a polysaccharide of N-acetylglucosamine, is a structural component of the sea louse exoskeleton [109]. Therefore, it is likely that the upregulation of *mbl2* is related to the recognition of *C. rogercresseyi* chitin, facilitating its identification and subsequent activation of innate mechanisms [110].

The regulation of granulocyte chemotaxis was shown to be increased in Atlantic salmon infested with *C. rogercresseyi*. The coordinated regulation of granulocyte chemotaxis and alternative pathway complement activation ensures an effective and localized immune response while minimizing damage to healthy host tissues [111,112,113]. A previous study reported that the chemotaxis of Atlantic salmon head kidney was significantly suppressed by inhibiting the complement system after *L. salmonis* infection [104]. Although the temperatures were similar between that report and our study, we suggest that the upregulation of complement and chemotaxis processes registered in our data could be related to the different sea louse species, tissue-specific responses, and the seasonal variability included in our study, together with the successful infestation in a natural productive environmental condition and the reduced and more productively realistic number of ectoparasites, which contrasts with the enormous number of copepodites used to infest Atlantic salmon in controlled laboratory conditions (50 copepodids/fish) [104].

In addition, we registered the killing of symbiont cells by the host. Among the genes associated with this biological process, our study found the upregulation of *cathelicidin antimicrobial peptide (camp)*, a family of antimicrobial peptides involved in the innate immune response that targets a broad spectrum of pathogens [114]. Importantly, previous reports suggest that CAMP plays a significant and complex role in the interaction between Atlantic salmon and sea lice [114]. In fact, the release of Cathelicidin from the skin of Atlantic salmon seems to trigger chemosensory neural activity in the marine parasitic copepod *L. salmonis*, alters their swimming behavior, and upregulates chemosensory-related genes [114]. Thus, the upregulation of camp suggests an action as an innate immunity component involved in the response against *C. rogercresseyi*; however, it could also have a side effect due to its action as an attractant, which would promote a potential increase in infestation in Atlantic salmon. Further studies are needed to verify the scope and consequences of this finding in *C. rogercresseyi* in spring but not in autumn.

**Figure 8:**
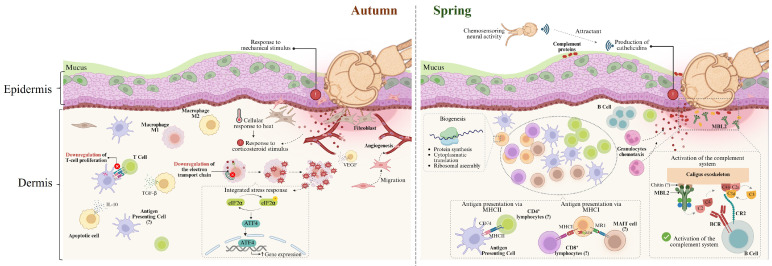
Suggested model of response for Atlantic salmon skin response to *C. rogercresseyi* infestation in autumn and spring.

The activation of the immune response triggers the activation of all cellular machinery associated with translational processes, promoting protein synthesis. In line with this idea, our study showed that the upregulation of processes associated with protein synthesis, such as cytoplasmic translation and ribosome assembly, may be related to the active production of proteins involved in the immune response [115]. The seasonal modulation of all these immune mediators has been reported elsewhere [116,117,118], thus reinforcing the idea that the skin’s response mechanisms to *C. rogercresseyi* infestation would be dependent on the seasonal temperature variation to which the fish are subjected throughout the production cycle. Taken together, these results reinforce the idea that the skin is a dynamic tissue [33], capable of immune recognition and with transcriptional machinery that enables it to respond to *C. rogercresseyi* infestation. A summary of the mechanism of response for Atlantic salmon skin response to C. rogercressey infestation in autumn and spring is detailed in Figure 8.

## 5. Conclusions

This study aimed to analyze the effect of the seasonal temperature on the skin transcriptome of Atlantic salmon infested with *C. rogercresseyi* in fjord-based farms in the Aysén Region during autumn and spring. The response in autumn was characterized by the perception of the infestation as a mechanical stimulus and the activation of the stress response. We propose that this physiological condition is responsible for the negative regulation of T-cell proliferation. We also identified the promotion of oxidative stress, which activates the integrated stress response and apoptosis as a mechanism for eliminating damaged cells. Additionally, we identified the modulation of genes associated with macrophage polarization in the context of an M2 phenotype. Thus, these biological processes are probably promoted as a strategy to restore cellular homeostasis by protecting the skin tissue cells from damage and oxidative stress caused by infestation with *C. rogercresseyi*.

By contrast, in the spring, we observed the activation of both innate and adaptive immune responses. For the innate immunity, we identified the promotion of the complement response. In this context, we also propose mechanisms associated with recognition and binding to specific carbohydrate patterns (like mannose and N-acetylglucosamine) that are structural components of the sea louse exoskeleton. At the innate level, we also observed the upregulation of *cathelicidin* as an innate component involved in the response against *C. rogercresseyi*; however, *cathelicidin* may also have a side effect due to its action as an attractant, which could potentially promote an increase in infestation in Atlantic salmon. We also registered the promotion of components linked with the processing and presentation of antigens, both in the context of MHC class I and class II. Importantly, we also suggest the participation of a subset of T cells called mucosal-associated invariant T (MAIT) cells against *C. rogercresseyi* infestation in Atlantic salmon skin. Additionally, we observed an upregulation of protein synthesis processes such as cytoplasmic translation and ribosome assembly, which may be related to the production of proteins involved in the immune response [115].

Taken together, the response in autumn would be characterized by a physiological condition of increased stress response, which would be responsible for compromising the activation of the immune response. In contrast, in spring, the innate immune response is activated as an adaptive immune response. However, the increase in *cathelicidin* as a mediator of the innate immune response would have an attractive effect on *C. rogercresseyi*, which would be an unwanted side effect on the development of protective mechanisms in the skin of Atlantic salmon against *C. rogercresseyi*.

The present study establishes the pioneering foundation for future research on Atlantic salmon against *C. rogercresseyi* infestation in open-sea cage farm environments. Although the results obtained comprise only the seasonal temperature variation for autumn and spring, future studies could consider the evaluation of the response to *C. rogercresseyi* in summer and winter as a representative reflection of the extreme temperatures to which Atlantic salmon are subjected throughout the annual temperature ramp.

## Figures and Tables

**Table 1:** Primers used for the validation of DEGs obtained from RNA-seq analysis by qPCR. The parameters and characteristics of the primers for the selected genes are summarized, indicating the full name, acronym, GenBank reference number for the gene nucleotide sequence, primer sequence (Fw: forward; Rv: reverse), amplicon size, primer efficiency, and the season in which the differential modulation of the gene is observed.

Gene Name	Acronym	GenBank No.	Primer Sequence ($5^{\prime }$–$3^{\prime }$)	Product Size (bp)	Primer Efficiency (%)	Season
*FOS like 1, AP-1 transcription factor subunit a*	*fosl1*	XM_014212300.2	Fw: AACCCTTCACCTTCCCAACC; Rv: CTGCGTCTCTCCAACTCCTC	125	106.7	autumn
*fos-related antigen 2*	*fosl2*	XM_014210013.2	Fw: AGTGTGCTCCTCTTCCGCT; Rv: CCGTCCTCATCGTCCTCTTG	103	100.6	autumn
*CCAAT Enhancer Binding Protein Beta*	*cebpb*	NM_001139913.1	Fw: GCATCCTCGTCTTCCTCCAG; Rv: TCGCTTCTTCCCCTTGACAC	108	106.6	autumn
*transcription factor AP-1*	*jun*	XM_014169347.2	Fw: AGGGCTTTGCGGAGGGATTT; Rv: GCTCACTGTCAAGGCGGAAAC	152	99.8	autumn
*tribbles pseudokinase 3*	*trib3*	XM_014167385.2	Fw: TCTCTTACGGACAAGCACGG; Rv: TAGCGACCCACCAGCAT	134	96.4	autumn
*dual specificity phosphatase 1*	*dusp1*	XM_014198502.2	Fw: TCTCTCAGGGGAGTATCAGTC; Rv: TGACGGCTTGGTACACATCT	196	108.8	autumn
*translocase of inner mitochondrial membrane 8 homolog A*	*timm8A*	XM_014199088.2	Fw: AAGGTTTCAGCAGTTGGTCC; Rv: CTCTACACAGTTCACAAAGC	117	111.1	autumn
*complement component 7b*	*c7*	XM_014134762.2	Fw: CACTACTTGTCAGAGGGGGC; Rv: GCTTCTTCTTCGTAACACACCG	124	105	spring
*heat shock protein 90, class B member 1*	*hsp90A1*	NM_001123532.1	Fw: GCTACCACAGTTCTCAGTCCG; Rv: TGCTCTCGCCAGTGATGT	105	100.2	spring
*CD74 molecule, major histocompatibility complex, class II invariant chain a*	*cd74*	XM_014199898.2	Fw: AAGGGTTTTGAGGCTTGGACAC; Rv: CTCGTCACACTGAGGGAGGTAG	162	107.3	spring
*probable ribosome biogenesis protein RLP24*	*rls24D1*	XP_013982426.1	Fw: GGCACAGTTCATCTTCAACAG; Rv: GCACCATCTTCTCCTCCATAACCTT	140	100.4	spring
*nucleolar RNA helicase 2*	*hfm1*	XM_014213631.2	Fw: TGTTGCCTAAGACTCCCAAGC; Rv: CTTTTTCCCCGTCACTGCC	102	98.2	spring
*collagen, type II, alpha 1b*	*col2A1*	NC_059453.1	Fw: CCTGGCGATACTGGTCCTCAA; Rv: AAGTCCCTTTTCTCCTCCCTTC	171	93.7	spring
*tenascin Ca (tnca)*	*tnc*	XM_014137401.2	Fw: AGATTGTTTTCACCCACCGC; Rv: CAGAAGCCAGAGTCACCAGT	157	104	spring
*cartilage intermediate layer protein, nucleotide pyrophosphohydrolase*	*cilp*	XM_014175441.2	Fw: TCTTTGTGTTGTCGTGCCTG; Rv: CTTGTGCCGTAGTGGTGACT	145	101	spring
*lipoprotein lipase*	*lpl*	NC_059455.1	Fw: AGTGACGGGAAGTTTGCTCA; Rv: GCTCTGGTGATGGGGGTAAC	196	100.7	spring
*adipocyte plasma membrane associated protein*	*apmap*	NM_001140255.1	Fw: TGAGCCGCCCCTTATGTCT; Rv: GCCGTGCCAGTGTAAATCAA	132	101.9	spring
*40s ribosomal protein*	*fau*	NM_001146588.1	Fw: TGCCCAGAACACTCACACC; Rv: CAGAGATGCCACAGTCCACC	164	101.9	reference gene
*Actin Beta*	*actb*	AF012125.1	Fw: ACTCAACCCCAAAGCCAACA; Rv: GCAGAGCGTAACCCTCGTAG	189	103.2	reference gene
*18S ribosomal RNA*	*18S*	AJ427629.1	Fw: AGGAATTGACGGAAGGGCAC; Rv: ACCAGACAAATCGCTCCACC	172	94	reference gene
*Eukaryotic Translation Elongation Factor 1 Alpha 1*	*ef1α-1*	NM_001141909.1	Fw: CTGGTGGTGTGGGTGAGTTT; Rv: AAACCGCTTCTGGCTGTAGG	152	98.9	reference gene

## Data Availability

RNA-Seq data are publicly available in the GEO database (NCBI). The original contributions presented in the study are included in the article/Supplementary Materials. Further inquiries can be directed to the corresponding author.

## Supplementary Materials

The following supporting information can be downloaded at https://www.mdpi.com/article/10.3390/ani15162369/s1: Table S1: Differentially expressed genes (DEGs); Table S2: Enriched biological processes in autumn; Table S3: Enriched biological processes in spring.
